# Effect of thoracic epidural anesthesia on postoperative outcome in major liver surgery: a retrospective cohort study

**DOI:** 10.1007/s00423-023-02900-w

**Published:** 2023-04-29

**Authors:** Christoph R. Behem, Juliane C. Wegner, Hans O. Pinnschmidt, Gillis Greiwe, Michael F. Graessler, Sandra Funcke, Rainer Nitzschke, Constantin J. C. Trepte, Sebastian A. Haas

**Affiliations:** 1 Department of Anesthesiology, Center of Anesthesiology and Intensive Care Medicine, University Medical Center Hamburg-Eppendorf, Martinistraße 52, 20246 Hamburg, Germany; 2 Department of Medical Biometry and Epidemiology, University Medical Center Hamburg-Eppendorf, Hamburg, Germany; 3 Department of Anesthesiology and Intensive Care Medicine, Rostock University Medical Center, Rostock, Germany

**Keywords:** Epidural anesthesia, Hepatectomy, Liver neoplasms, Perioperative care, Pain management

## Abstract

**Purpose:**

Postoperative complications after major liver surgery are common. Thoracic epidural anesthesia may provide beneficial effects on postoperative outcome. We strove to compare postoperative outcomes in major liver surgery patients with and without thoracic epidural anesthesia.

**Methods:**

This was a retrospective cohort study in a single university medical center. Patients undergoing elective major liver surgery between April 2012 and December 2016 were eligible for inclusion. We divided patients into two groups according to whether or not they had thoracic epidural anesthesia for major liver surgery. The primary outcome was postoperative hospital length of stay, i.e., from day of surgery until hospital discharge. Secondary outcomes included 30-day postoperative mortality and major postoperative complications. Additionally, we investigated the effect of thoracic epidural anesthesia on perioperative analgesia doses and the safety of thoracic epidural anesthesia.

**Results:**

Of 328 patients included in this study, 177 (54.3%) received thoracic epidural anesthesia. There were no clinically important differences in postoperative hospital length of stay (11.0 [7.00–17.0] vs. 9.00 [7.00–14.0] days, *p* = 0.316, primary outcome), death (0.0 vs. 2.7%, *p* = 0.995), or the incidence of postoperative renal failure (0.6 vs. 0.0%, *p* = 0.99), sepsis (0.0 vs. 1.3%, *p* = 0.21), or pulmonary embolism (0.6 vs. 1.4%, *p* = 0.59) between patients with or without thoracic epidural anesthesia. Perioperative analgesia doses — including the intraoperative sufentanil dose (0.228 [0.170–0.332] vs. 0.405 [0.315–0.565] μg·kg^−1^·h^−1^, *p* < 0.0001) — were lower in patients with thoracic epidural anesthesia. No major thoracic epidural anesthesia-associated infections or bleedings occurred.

**Conclusion:**

This retrospective analysis suggests that thoracic epidural anesthesia does not reduce postoperative hospital length of stay in patients undergoing major liver surgery — but it may reduce perioperative analgesia doses. Thoracic epidural anesthesia was safe in this cohort of patients undergoing major liver surgery. These findings need to be confirmed in robust clinical trials.

**Supplementary Information:**

The online version contains supplementary material available at 10.1007/s00423-023-02900-w.

## Introduction

Liver resections are major surgeries with high rates of postoperative complications [1–3]. To reduce postoperative complications and enhance recovery after surgery, attempts to implement standardized interdisciplinary concepts have been made [4]. Whether thoracic epidural anesthesia should be routinely used in patients undergoing hepatic resection surgery remains controversial. On the one hand, thoracic epidural anesthesia potentially helps control pain and reduce intraoperative opioid doses [5]. Additionally, thoracic epidural anesthesia may mitigate systemic inflammation and improve coagulation, pulmonary function, and intestinal perfusion and motility [6, 7]. On the other hand, thoracic epidural anesthesia itself can cause complications. Especially in cirrhotic patients with impaired coagulation or in patients undergoing expanded liver resection, thoracic epidural anesthesia bears the risk of bleeding and epidural hematoma [8–10]. Only few studies investigated effects of thoracic epidural anesthesia on hospital length of stay and postoperative complications in patients undergoing liver surgery — and they revealed contrasting results [11–15].

In our institution, thoracic epidural anesthesia was routinely used for liver resection from January 2012 to June 2015, but was not routinely used thereafter. We aimed to investigate effects of thoracic epidural anesthesia on postoperative outcomes in patients undergoing major liver surgery. Specifically, we performed a retrospective cohort study to test the primary hypothesis that postoperative hospital length of stay is shorter in major liver surgery patients with than in patients without thoracic epidural anesthesia. Additionally, we investigated the effect of thoracic epidural anesthesia on 30-day postoperative mortality, major postoperative complications, and perioperative analgesia doses, and the safety of thoracic epidural anesthesia.

## Material and methods

### Ethics

The ethics committee approved the study and waived the need to obtain informed consent for collection, analysis, and publication of data for this retrospective cohort study (Name and address of ethics committee: Ethikkommission der Ärztekammer Hamburg, Weidestraße 122 b, 22083 Hamburg, Germany; President of ethics committee: Prof. Dr. med. M. Carstensen; Process No. WF-022/17; Date of approval: 04/26/2017).

### Study design

This was a retrospective observational cohort study at the University Medical Center Hamburg-Eppendorf (Hamburg, Germany) to investigate the effect of thoracic epidural anesthesia on postoperative outcomes in patients undergoing major liver surgery.

### Inclusion and exclusion criteria

Adult patients undergoing elective major liver surgery (hemi-hepatectomy, atypical liver resection, or biliodigestive anastomosis as well as bile duct revisions and surgical treatment of liver cysts) between April 1, 2012, and December 31, 2016, were eligible for study inclusion. We aimed to include approximately 150 patients per group. Exclusion criteria were laparoscopic liver surgery, explorative laparotomy, and redo procedures. Further, patients after liver transplantation or patients undergoing combined surgery for different organs were not included. In addition, we excluded patients if the anesthesia records or medical charts were incomplete.

### Exposure

Patients were divided into two groups according to whether or not they had thoracic epidural anesthesia for major liver surgery. Thoracic epidural anesthesia was used per institutional routine since January 2012 (unless there were contraindications). In June 2015, anesthesiologists and surgeons agreed to stop routinely using thoracic epidural anesthesia in patients undergoing major liver surgery. This decision was based on reservations regarding the risk of bleeding associated with the use of thoracic epidural anesthesia in major liver surgery. In patients with thoracic epidural anesthesia, intraoperative thoracic epidural bolus injections of 0.25% bupivacaine with sufentanil 0.75 μg·ml^−1^ were used and repeated every 60–120 min, followed by postoperative continuous thoracic epidural anesthesia (6–8 ml·h^−1^ bupivacaine 0.125% with sufentanil 0.75 μg·ml^−1^ for patients < 70 years of age, 6–8 ml·h^−1^ bupivacaine 0.125% for patients ≥ 70 years of age). In addition, a patient-controlled bolus application of 2 ml was allowed every 15 min if needed.

### Primary outcome

The primary outcome was postoperative hospital length of stay, i.e., from day of surgery until hospital discharge.

### Secondary outcomes

Secondary outcomes were censored at postoperative day 30 and included postoperative complications (bile leakage, renal failure, sepsis, pulmonary arterial embolism, thrombosis of inferior cava vein, and pulmonary infiltrates or congestion on chest x-ray), intensive care unit length of stay, 30-day postoperative mortality, postoperative laboratory findings (liver and kidney values, coagulation profiles, and inflammation parameters), perioperative analgesia doses, and the safety of thoracic epidural anesthesia (occurrence of dura perforations, epidural infections, epidural abscess formation, epidural hematoma, or nerve injuries after epidural catheter placement or removal).

### Data collection

Data were extracted from electronic health and anesthesia records (Soarian ® Clinicals, and Soarian ® Health Care Archive, Cerner Deutschland GmbH, Idstein, Germany).

### Statistical analysis

Descriptive statistics are presented as median and interquartile range [IQR] for continuous variables and as total numbers and percentage for categorical variables. We compared baseline variables, preoperative laboratory findings, and perioperative variables, between patients with and without thoracic epidural anesthesia using Mann–Whitney *U* tests (continuous variables) and Fisher’s exact (categorical variables). Multivariate analyses were used to compare primary outcome, postoperative complications, and 30-day postoperative mortality between patients with and without thoracic epidural anesthesia. In these multivariate analyses (binary logistic regression, Cox regression, or general linear modelling, depending on whether the outcome variable was binary, time-to-event, or continuous), the primary independent variable thoracic epidural anesthesia was always adjusted for coronary artery disease, diabetes mellitus, and preoperative diuretics — because these covariates had shown significant relations to thoracic epidural anesthesia. Other covariates were only included in the multivariate analyses if they were statistically significant. For the categorial outcomes postoperative renal failure, pulmonary embolism, pulmonary congestion, bile leakage, and thrombosis of inferior cava vein, the number of events were too few to render multivariate analysis feasible, since the number of included covariates would have been as high or even higher than the number of outcome events. For these outcomes, group comparisons were done by Fisher’s exact tests. As baseline laboratory values differed substantially between patients with and without thoracic epidural anesthesia, we also included baseline laboratory values as covariates for multivariate comparison of postoperative laboratory findings. For creatinine, international normalized ratio of prothrombin time (INR), and partial thromboplastin time (PTT), we also found significant effects for coronary artery disease which was included as covariable as well. The *p* values of two-tailed tests are presented. Since the nature of this study is explorative, no a priori sample size calculation was performed and no *α* error adjustments for multiple comparisons were made and two-tailed *p* values < 0.05 were considered statistically significant. All analyses were performed using SPSS v. 25 (IBM © Inc., Armonk, NY, USA).

## Results

We screened 368 patients who met the inclusion criteria, but excluded 42 because they had redo surgery. For patients with thoracic epidural anesthesia, a time period between April 2012 and January 2014 was sufficient, and for patients without epidural anesthesia, a time period between April 2012 and February 2016 was needed to include a sufficient number of patients.

We thus analyzed data of 326 patients. One hundred seventy-seven of these 326 patients (54.3%) had thoracic epidural anesthesia (Table 1, Table 2). Patients without thoracic epidural anesthesia more often had coronary artery disease and diabetes mellitus and more often were treated with diuretics before surgery than patients with thoracic epidural anesthesia.

**Table 1:** Baseline values

	Patients with thoracic epidural anesthesia	Patients without thoracic epidural anesthesia	
Baseline values	*n* (%)/median [IQR]	*n* (%)/median [IQR]	*p* value
Number of patients (%)	177 (54.3)	149 (45.7)	
Age (years)	63.0 [54.0–71.0]	64.0 [53.0–73.0]	0.642
Gender (%)			0.820
Male	106 (59.9)	92 (61.7)	
Female	71 (40.1)	57 (38.3)	
Body mass index (kg m^−2^)	25.1 [22.2–28.5]	25.3 [22.4–28.1]	0.998
Diagnosis (%)			0.075
Hepatocellular carcinoma	30 (16.9)	38 (25.7)	
Adenoma	9 (5.1)	10 (6.8)	
Cholangiocarcinoma	46 (26.0)	29 (19.6)	
Metastatic liver disease	60 (33.9)	36 (24.3)	
Other cause	32 (18.1)	35 (23.6)	
Type of surgery (%)			0.538
Atypical resection	95 (53.7)	84 (56.4)	
Hemi-hepatectomy	74 (41.8)	55 (36.9)	
Other	8 (4.5)	10 (6.7)	
Liver disease (%)			0.333
Steatosis	5 (2.8)	5 (3.4)	
Cirrhosis	16 (9.0)	21 (14.1)	
Comorbidities (%)			
Coronary artery disease	8 (4.5)	21 (14.1)	0.003
Atrial fibrillation	17 (9.6)	17 (11.4)	0.717
Heart failure	11 (6.2)	13 (8.8)	0.401
Renal failure	9 (5.1)	7 (4.7)	0.999
Diabetes mellitus	24 (13.6)	35 (23.5)	0.030
Peripheral artery disease	3 (1.7)	3 (2.0)	0.999
History of stroke	16 (9.0)	7 (4.7)	0.136
Chronic obstructive pulmonary disease	7 (4.0)	8 (5.4)	0.602
Obstructive sleep apnea	2 (1.1)	1 (0.7)	0.999
Preoperative medication (%)			
Beta-blockers	40 (22.6)	34 (23.0)	0.999
ACE-inhibitor/AT_1_ receptor antagonists	46 (26.0)	33 (22.3)	0.516
Diuretics	12 (6.8)	22 (14.9)	0.028
Phenprocoumon	5 (2.8)	6 (4.1)	0.555
Opioids	9 (5.1)	8 (5.4)	0.999
Nonsteroidal anti-inflammatory drugs	4 (2.3)	2 (1.4)	0.692

Baseline values for different groups. Data are presented as median [IQR] for metric variables as well as total number (percentage) for categorical variables. *ACE*, angiotensin converting enzyme; *AT*_*1*_*-receptor*, angiotensin-II receptor type 1

**Table 2:** Preoperative laboratory findings

	Patients with thoracic epidural anesthesia	Patients without thoracic epidural anesthesia	
Preoperative laboratory findings	Median [IQR]	Median [IQR]	*p* value
Hemoglobin (g·dl^−1^)	13.3 [12.3–14.6]	13.1 [11.8–14.3]	0.199
Leucocytes (bil·l^−1^)	7.50 [6.15–9.05]	6.95 [5.50–8.40]	0.006
Thrombocytes (bil·l^−1^)	271 [215–366]	241 [114–184]	<0.001
Bilirubin (mg·dl^−1^)	0.500 [0.400–0.600]	0.500 [0.400–0.900]	0.097
Creatinine (mg·dl^−1^)	0.900 [0.700–1.00]	0.900 [0.800–1.00]	0.096
AST (U·l^−1^)	29.5 [20.5–44.5]	32.5 [21.0–62.5]	0.152
GGT (U·l^−1^)	119.0 [45.0–214.0]	99.0 [44.0–310.0]	0.550
CRP (mg·l^−1^)	6.00 [4.00–22.0]	4.00 [4.00–14.0]	0.036
INR	0.98 [0.940–1.04]	1.01 [0.970–1.08]	<0.001
PTT (s)	29.5 [27.2–31.4]	29.6 [27.9–32.8]	0.063
Fibrinogen (g·l^−1^)	4.32 [3.58–5.92]	4.02 [3.18–5.12]	0.007

Baseline laboratory findings for different groups. Data are presented as median [IQR]. *AST*, aspartate transaminase; *CRP*, C-reactive protein; *GGT*, gamma-glutamyl-transferase; *INR*, international normalized ratio of prothrombin time; *PTT*, partial thromboplastin time

There was no clinically important difference in postoperative hospital length of stay between patients with and without thoracic epidural anesthesia (11.0 [7.00–17.0] vs. 9.00 [7.00–14.0] days, *p* = 0.316).

None of the 177 patients with thoracic epidural anesthesia but 4 of 149 patients without thoracic epidural anesthesia died within 30 days of surgery (*p* = 0.995). There were no clinical important differences in postoperative complications between patients with and without thoracic epidural anesthesia (Table 3).

**Table 3:** Secondary outcome measures

	Patients with thoracic epidural anesthesia	Patients without thoracic epidural anesthesia	
Secondary outcome measures	*n* (%)/median [IQR]	*n* (%)/median [IQR]	*p* value
Death (%)	0 (0.0)	4 (2.7)	0.995
Intensive care unit length of stay (days)	1.00 [1.00–2.00]	1.00 [1.00–1.00]	0.047
Sepsis (%)	0 (0.0)	2 (1.3)	0.208
Renal failure (%)	1 (0.6)	0 (0.0)	0.999
Pulmonary embolism (%)	1 (0.6)	2 (1.4)	0.593
Thrombosis inferior cava vein (%)	0 (0.0)	1 (0.7)	0.457
Pulmonary infiltrates (%)	4 (2.3)	3 (2.1)	0.999
Pulmonary congestion (%)	0 (0.0)	1 (0.7)	0.378
Bile leakage (%)	2 (1.1)	0 (0.0)	0.502
Defecation prior postoperative day 2 (%)	11 (6.3)	15 (10.1)	0.224

Secondary outcome measures for different groups. Data are presented as median [IQR] for metric variables as well as total number (percentage) for categorical variables

Patients with thoracic epidural anesthesia had lower total doses of sufentanil compared to patients without thoracic epidural anesthesia (0.228 [0.170–0.332] vs. 0.405 [0.315–0.565] μg·kg^−1^·h^−1^, *p* < 0.0001), a difference that is clinically important. Moreover, piritramide doses as well as the proportions of patients who received metamizole, as well as clonidine, were lower among patients with than without thoracic epidural anesthesia (Table 4). Patients with thoracic epidural anesthesia received higher doses of colloids (2.23 [0.000–4.59] vs. 1.24 [0.000–3.65] ml·kg^−1^·h^−1^, *p* = 0.025).

**Table 4:** Perioperative parameters

	Patients with thoracic epidural anesthesia	Patients without thoracic epidural anesthesia	
Perioperative parameters	*n* (%)/median [IQR]	*n* (%)/median [IQR]	*p* value
Duration of anesthesia induction (min)	55.0 [45.0–60.0]	40.0 [30.0–50.0]	<0.0001
Duration of surgery (min)	230 [160–285]	210 (145–295]	0.223
Duration of PACU stay (min)	75.0 [55.0–102.5]	70.0 [50.0–90.0]	0.136
Sufentanil (μg·kg^−1^·h^−1^)	0.228 [0.170–0.332]	0.405 [0.315–0.565]	<0.0001
Piritramide (mg)	0.000 [0.000–0.000]	0.000 [0.000–7.50]	<0.001
Application of metamizole (%)	53 (29.9)	75 (50.7)	<0.001
Application of clonidine (%)	3 (1.7)	21 (14.5)	<0.0001
Crystalloids (ml·kg^−1^·h^−1^)	7.24 [5.12–10.3]	7.34 [4.54–10.6]	0.938
Colloids (ml·kg^−1^·h^−1^)	2.23 [0.000–4.59]	1.24 [0.000–3.65]	0.025
Red blood cells (*n*)	0.000 (0.000–0.000]	0.000 [0.000–0.000]	0.504
Fresh frozen plasma (*n*)	0.000 [0.000–0.000]	0.000 [0.000–0.000]	0.007
Maximum dose of norepinephrine (μg·kg^−1^·min^−1^)	0.142 [0.100–0.209]	0.120 [0.079–0.188]	0.017
Need for oxygen during PACU stay (%)	141 (84.4)	123 (90.4)	0.167

Perioperative parameters for different groups. Data are presented as median [IQR] for metric variables as well as total number (percentage) for categorical variables. *PACU*, post-anesthesia care unit

In patients with thoracic epidural anesthesia, anesthesia induction took 15 min longer than in patients without thoracic epidural anesthesia (55.0 [5.0–60.0] vs. 40.0 [30.0–50.0] min, *p* < 0.0001), a difference that might be of clinical and economic importance. There were no clinical important differences in laboratory findings between patients with and without thoracic epidural anesthesia, except for C-reactive protein levels, which were higher among patients with than in patients without thoracic epidural anesthesia (Table 5).

**Table 5:** Difference of laboratory findings on postoperative day 3

	Patients with thoracic epidural anesthesia	Patients without thoracic epidural anesthesia	
Difference of laboratory findings on postoperative day 3	Median [IQR]	Median [IQR]	*p* value
Hemoglobin (g·dl^−1^)	−2.90 [(−4.20)–(−1.50)]	−2.60 [(−4.30)–1.30]	0.489
Leucocytes (bil·l^−1^)	1.40 [(−0.20)–3.30]	1.00 [(−0.60)–2.80]	0.171
Thrombocytes (bil·l^−1^)	−65.0 [(−121.0)–(−24.0)]	−39.0 [(−88.0)–(−5.00)]	0.684
Bilirubin (mg·dl^−1^)	0.100 [0.000–0.300]	0.100 [(−0.100)–0.300]	0.255
Creatinine (mg·dl^−1^)	−0.100 [(−0.200)–0.000]	−0.100 ((−0.200)–0.000]	0.804
AST (U·l^−1^)	40.5 [11.5–90.0]	37.0 [1.00–92.0]	0.321
GGT (U·l^−1^)	−21.0 [(−76.0)–8.00]	−9.00 [(−113–20.5]	0.927
CRP (mg·l^−1^)	91.5 [47.5–135.0]	72.0 [35.5–112.5]	0.002
INR	0.040 [(−0.020)–0.140]	0.010 [(−0.030)–0.070]	0.046
PTT (s)	4.20 [1.20–8.20]	5.99 [(−0.20)–6.90]	0.005
Fibrinogen (g·l^−1^)	1.34 [(−0.25)–3.03]	1.58 [0.28–2.94]	0.253

Difference of laboratory findings on postoperative day 3 for different groups. Data are presented as median [IQR]. AST, aspartate transaminase; CRP, C-reactive protein; GGT, gamma-glutamyl-transferase; INR, international normalized ratio of prothrombin time; PTT, partial thromboplastin time

Dura perforations occurred in 1.7% of patients with thoracic epidural anesthesia. The rate of intraoperative thoracic epidural bolus injection was 9.68 [7.81–11.5] ml·h^−1^. The duration of postoperative thoracic epidural anesthesia use was 5.0 [3.0–6.0] days. No patients had epidural infections, epidural abscess formation, epidural hematoma, or nerve injuries after epidural catheter placement or removal.

## Discussion

In this retrospective cohort study, we revealed that thoracic epidural anesthesia does not reduce hospital length of stay in patients undergoing major liver surgery — but it may reduce required analgetic doses.

Thoracic epidural anesthesia may provide beneficial effects on postoperative outcome in patients undergoing major abdominal surgery [16]. In liver surgery, beneficial effects must be weighed against the increased risk of bleeding following liver resection, which may lead to increased complication rates associated with thoracic epidural anesthesia. Balance between beneficial effects and risks are scarcely investigated. Whether beneficial effects of thoracic epidural anesthesia in liver surgery translate into shorter hospital lengths of stay and less postoperative complications remains unknown. In our retrospective analysis, postoperative hospital length of stay did not differ between patients with and without thoracic epidural anesthesia. Moreover, there were no differences in intensive care unit length of stay between patients with and without thoracic epidural anesthesia. In line with our findings, a retrospective comparison of epidural anesthesia with intravenous patient-controlled analgesia in 226 patients for live liver donation also did not show a difference in hospital length of stay [17]. In contrast to our results, a retrospective analysis of 126 cirrhotic patients undergoing liver surgery in combination with a fast-track protocol demonstrated a shorter hospital length of stay in patients with epidural anesthesia than in patients without epidural anesthesia [11]. On the contrary, a retrospective matched cohort study revealed a longer hospital length of stay for patients receiving epidural anesthesia for liver surgery while a retrospective analysis of > 20,000 patients undergoing hepatopancreatic surgery also showed an increase in hospital length of stay in patients with epidural anesthesia. However, it should be mentioned that the proportion of patients having epidural anesthesia was considerably low in both studies [2, 12].

With regard to postoperative complications, in our study, rates of pulmonary or renal complications, bile leakage, and sepsis were similar between patients with and without thoracic epidural anesthesia. However, the complication rates were low and the number of patients thus may be insufficient to definitely explore the effect of thoracic epidural anesthesia on postoperative complications. Nevertheless, in line with our results, a retrospective analysis of 177 liver surgery patients found no differences in postoperative complications — although complication rates were considerably higher than in our study [18]. In a retrospective study comparing epidural anesthesia with patient-controlled analgesia following hepatectomy, overall postoperative outcomes were also similar between groups [19]. In contrast, a retrospective analysis in 829 patients having liver resections with and without epidural anesthesia revealed a potential risk for postoperative acute kidney injury associated with epidural anesthesia [20]. A recent propensity score-matched analysis showed no differences in hospital length of stay and postoperative complications between patients having liver surgery with and without epidural anesthesia — but an improvement in 1-year survival for patients receiving epidural anesthesia [14].

Epidural anesthesia provides beneficial effects on perioperative analgesia [2, 13, 17, 21]. It has been shown to provide effective postoperative analgesia in liver surgery [5, 19, 22]. In our study, intraoperative sufentanil doses were lower for patients with compared to patients without thoracic epidural anesthesia. Moreover, there were lower proportions of patients receiving non-opioid analgesics (metamizole) or co-analgesics (clonidine) in patients with compared to patients without epidural anesthesia. In this context, the increased duration of anesthesia duration attributed to epidural anesthesia would be inside an acceptable range for most physicians.

Sympathetic block induced by thoracic epidural anesthesia may lead to hypotension requiring administration of fluids or vasopressors. Higher rates of hypotension and higher amounts of administered fluids as well as vasopressors in patients with epidural anesthesia were previously reported [18, 23]. This may be in line with our study showing a higher amount of colloids in patients with compared to patients without thoracic epidural anesthesia. However, the amount of crystalloids was not different between patents with compared to patients without thoracic epidural anesthesia and we did not find clinical important differences of vasopressor doses. Furthermore, we could not find a higher proportion of renal failure, pulmonary congestion, or other complications in patients with thoracic epidural anesthesia that could be attributed to hypotension or negative effects of potentially increased fluid requirements.

Sympatholytic effects of thoracic epidural anesthesia may lead to improvements of intestinal motility. We retrospectively analyzed occurrence of defecation prior the second postoperative day and could not find advantages for patients with thoracic epidural anesthesia. In line with our study, no advantages of epidural anesthesia after liver surgery in regard to vomiting or first fluid intake were reported [17]. In contrast, a Cochrane meta-analysis revealed a high quality of evidence for acceleration of gastrointestinal transit after major surgery when epidural anesthesia was used [24]. Beneficial effects of thoracic epidural anesthesia may have possibly been found for our patient collective undergoing major liver surgery by use of different parameters of gastrointestinal function.

A major concern when using epidural anesthesia in liver surgery is increased risks of bleeding due to a changed postoperative coagulation function [25]. Reductions in coagulation *prior* liver surgery may preclude the use of epidural anesthesia. In our patient collective, neuraxial anesthesia was performed in accordance with the actual guidelines precluding patients with impaired coagulation or existing use of anticoagulants or antiplatelet drugs within certain time intervals prior surgery. Moreover, changes in coagulation profile including thrombocytopenia are frequently observed *after* liver resection due to perioperative blood loss or impairments of liver function [8, 26, 27]. Concerns about the risk of epidural hematoma or prolonged duration of indwelling catheters caused by coagulation derangements have been raised in this regard. Safety issues for use of epidural anesthesia in liver surgery have been proposed. However, safe use of epidural anesthesia has been shown despite coagulation changes after liver resection [5, 9, 25, 26, 28]. Although some authors reported delayed withdrawal of epidural catheters caused by coagulation derangements, this has not led to the formation of epidural hematoma or abscess [8, 18]. In our study, there were no important changes of coagulation profile in the postoperative period between patients with and patients without thoracic epidural anesthesia, although thrombocytes were reduced in both groups. Moreover, there was no incidence of epidural hematoma, epidural abscess, or nerve injuries. Therefore, our study supports the safe use of epidural anesthesia in major liver surgery. Nevertheless, given the very low overall incidence of epidural hematoma [29, 30], robust prospective studies are needed to adequately address this issue.

Epidural anesthesia itself can affect coagulation. Reduction of thrombotic events has been shown for epidural anesthesia leading to improvements of outcome. While the mechanisms are not fully understood, they include influences on coagulation, fibrinolysis, inflammation, and platelet aggregation [7]. As mentioned, there were no clinical important differences in thrombocytes and coagulation profile between patients with and without thoracic epidural anesthesia. Moreover, in regard to thrombotic risks, there were no differences in the occurrence of inferior vena cava thrombosis or pulmonary embolism between patients with and patients without thoracic epidural anesthesia.

In our retrospective analysis, four patients died within 30 days. Causes of death were respiratory failure in one case, sepsis in two cases, and thrombosis of hepatic vein in one case. Although all of them were in the group of patients without epidural anesthesia, multivariate analysis did not reveal a significant effect between groups. In addition, this was a retrospective study and mortality was included only as secondary outcome variable. Prospective studies with a higher patient number are needed to address potential beneficial effects of thoracic epidural anesthesia on postoperative mortality after major liver surgery in the future.

Recently, a number of studies have compared effects of epidural anesthesia with local wound infiltration techniques in patients undergoing major liver surgery [23, 31, 32]. In our study, no patient received local anesthesia techniques and the comparison between local and epidural anesthesia is beyond the scope of this study. While there has been a huge number of studies addressing the effect of epidural anesthesia on hospital length of stay and postoperative complications in major surgery [24, 33, 34], our analysis was restricted to patients having major liver surgery and therefore is not generalizable to other types of surgery — specifically thoracic and gastrointestinal surgery. However, we deliberately focused on liver surgery to gather insights on the balance between potential benefits and the specific risks associated with epidural anesthesia for this patient collective. As further limitation, it should be considered that apart from analgesia use in the perioperative period, we did not assess additional parameters of analgesia including total postoperative analgesia consumption, subjective values like numeric rating scale scores, postoperative nausea, or endocrine parameters. In addition, rates of complication were generally low for both groups possibly reducing the ability to show differences of outcome parameters for this patient collective. In our institution, thoracic epidural anesthesia was routinely used for liver resection from January 2012 to June 2015, but was not used thereafter. This decision was based on reservations about potential bleeding risks that may be associated with the use of thoracic epidural anesthesia in major liver surgery. Thus, indication for thoracic epidural anesthesia was only secondary based on comorbidities or surgical extent minimizing selection bias. The chosen time interval was intended to reduce selection bias and most baseline values were comparable between groups. Nonetheless, concomitant diseases were not comparable for all parameters between groups, thus representing potential sources of confounding. Therefore, as described in the statistics section, we performed multivariate analysis and included coronary artery disease, diabetes mellitus, and application of diuretics as covariables because they had shown significant associations with use of thoracic epidural anesthesia (data not shown). However, for the categorial outcome variables perioperative renal failure, pulmonary embolism, pulmonary congestion, bile leakage, and thrombosis of inferior cava vein, the number of events were too few to render multivariate analysis feasible as described in the statistics section. Although this may have reduced potential bias, our results should be confirmed in robust prospective trials.

## Conclusion

In conclusion, this retrospective analysis suggests that thoracic epidural anesthesia does not reduce hospital length of stay in patients undergoing major liver surgery — but it may reduce perioperative analgesia doses. Thoracic epidural anesthesia did not lead to important differences in postoperative complications and was safe in this cohort of patients undergoing major liver surgery. These findings need to be confirmed in robust clinical trials.

### Supplementary information

Supplementary file 1 Strobe Statement—Checklist of items that should be included in reports of cohort studies

## Data Availability

Original data will be provided upon reasonable request.

## Abbreviations

ACE: angiotensin converting enzyme
AST: aspartate transaminase
AT_1_-receptor: angiotensin-II receptor type 1
CRP: C-reactive protein
GGT: gamma-glutamyl-transferase
INR: international normalized ratio of prothrombin time
PACU: post-anesthesia care unit
PTT: partial thromboplastin time
